# Diagnostic Value of Color Doppler Ultrasound Combined with Superb Microvascular Imaging in the Detection of Small Renal Tumors Less than 3 cm Treated with Jinkui Shenqi Pills

**DOI:** 10.1155/2021/5327331

**Published:** 2021-09-03

**Authors:** Yuping Gong, Shuhui Li

**Affiliations:** ^1^Department of Ultrasonic Imaging, Traditional Chinese Medicine Hospital of China Three Gorges University, Yichang Traditional Chinese Medicine Hospital, Yichang 443000, Hubei Province, China; ^2^Department of Nephropathy, The People's Hospital Attached to Sanxia (Three Gorges) University, The First Hospital of Yichang, Yichang 443000, Hubei Province, China

## Abstract

The purpose of this study was to investigate the diagnostic value of color Doppler ultrasound combined with superb microvascular imaging (SMI) in the detection of small renal tumors less than 3 cm treated with Jinkui Shenqi pills. 50 cases were randomly selected from the patients with angioleiomyoma (a kind of small renal tumor) less than 3 cm confirmed by pathological examination and treated in our hospital from January 2018 to January 2020. All patients were treated with Jinkui Shenqi pills. All patients were first detected by color Doppler ultrasound and then by SMI. The results of color Doppler ultrasound were used as the control group, while those of color Doppler ultrasound combined with SMI were used as the experimental group. After that, the specificity, sensitivity, positive and negative detection results, and detection accuracy were compared between the two groups. The specificity and sensitivity in the experimental group were significantly higher than those in the control group, with statistical significance (*P* < 0.05). The cases of positive and negative detection results in the experimental group were significantly higher than those in the control group, with statistical significance (*P* < 0.05). The detection accuracy in the experimental group was significantly higher than that in the control group, with statistical significance (*P* < 0.05). The specificity, sensitivity, positive and negative detection results, and detection accuracy of color Doppler ultrasound combined with SMI in the detection of small renal tumors less than 3 cm treated with Jinkui Shenqi pills were all significantly higher than those of color Doppler ultrasound; therefore, the application of color Doppler ultrasound combined with SMI for the diagnosis of small renal tumors is of high value.

## 1. Introduction

Small renal tumors including benign and malignant tumors are very common urinary diseases. The diameter of small renal tumors is generally no more than 3 cm at the early stage when the patients may not suffer from symptoms such as low back pain, odynuria, and hematuria, with little effect on their daily life. Therefore, it is highly likely that the early diagnosis and treatment will be missed, resulting in the aggravation of the patients' conditions and seriously affecting patients' life health [1–3]. Jinkui Shenqi pills are a kind of Chinese traditional medicine (CTM) mainly to treat edema due to kidney deficiency, soreness and weakness of waist and knees, and other symptoms with the function of warming and invigorating kidney yang. The clinical symptoms of patients with small renal tumors can be alleviated to a certain extent after the treatment with Jinkui Shenqi pills. Color Doppler ultrasound is commonly used in the clinical diagnosis of small renal tumors. However, due to the small size of small renal tumors, the missed diagnosis and misdiagnosis may occur with single color Doppler ultrasound, adversely affecting patients' timely treatment [4–6]. Superb microvascular imaging (SMI) is a new type of imaging technology that detects human blood flow and displays the flow in a visual form, which can diagnose the vascular diseases of patients directly and accurately. It has been reported that the sensitivity and specificity of SMI in the detection and diagnosis of small renal tumors are relatively high, which can greatly improve the detection rate and accuracy of small renal tumors [7–9]. To further study the application value of color Doppler ultrasound combined with SMI in the diagnosis of small renal tumors, patients with small renal tumors were selected as the research objects to compare the specificity, sensitivity, positive and negative detection results, and detection accuracy between color Doppler ultrasound and SMI combined with color Doppler ultrasound, aiming to clarify the application value and diagnostic effect of color Doppler ultrasound combined with SMI in clinical practice, with details reported as below.

## 2. Materials and Methods

### 2.1. General Information

50 cases were randomly selected from the patients with angioleiomyoma (a kind of small renal tumor) less than 3 cm confirmed by pathological examination and treated in our hospital from January 2018 to January 2020. All patients were first detected by color Doppler ultrasound and then by superb microvascular imaging (SMI). The results of color Doppler ultrasound were used as the control group, while those of color Doppler ultrasound combined with SMI were used as the experimental group. The patients were aged 22–69 years, with an average age of 46.22 ± 5.89 years, an average weight of 69.95 ± 4.70 kg, an average height of 168.82 ± 5.79 cm, and an average disease course of 3.20 ± 0.59 months.

### 2.2. Inclusion/Exclusion Criteria

#### 2.2.1. Inclusion Criteria

  ① The patients had the clinical manifestation of angioleiomyoma (a kind of small renal tumor)
  ② The patients had normal heart and lung functions
  ③ The patients had no congenital diseases
  ④ The patients had no drug allergy history, drug abuse history, and bad addiction
  ⑤ This study was approved by the Hospital Ethics Committee, and the patients all voluntarily participated in the study and signed informed consent

#### 2.2.2. Exclusion Criteria

  ① The patients had coagulation disorder or were taking anticoagulants
  ② The patients' tumors were more than 3 cm in diameter
  ③ The patients had disturbance of consciousness and could not cooperate with this study

### 2.3. Methods

#### 2.3.1. Detection Methods

All patients were first detected by color Doppler ultrasound and then by superb microvascular imaging (SMI). The results of color Doppler ultrasound were used as the control group, while those of color Doppler ultrasound combined with SMI were used as the experimental group. At 1 hour before detection, patients drank enough water to suppress urine and keep the bladder in a filling state. A color Doppler ultrasound detector (Toshiba Aplio 400 with the probe model as PLT-1005BT) was adopted. With patients taking supine positions during detection, the medical staff moved the probe to scan patients' kidney areas, with the probe frequency of 3.0–7.0 MHz, and recorded the location and size of lesions or tumors [10–12]. After the detection by color Doppler ultrasound, the detector was adjusted to the CDFI, PD, and ADF modes with the color SMI mode. When the patients held their breath, SMI scanning was performed with the lesions as the center and samples selected in the area of 1 cm around the lesions. After the adjustment of color gain and the disappearance of artifact without any factors affecting the diagnostic results, the microvessels were clearly visible to determine the diagnostic results.

In this study, the detection was performed, and the detection results were read by two attending physicians with more than 7 years of experience in abdominal ultrasound examination. If there were any differences, agreement would be reached after the results were discussed.

#### 2.3.2. Treatment Methods

All patients orally took Jinkui Shenqi pills (manufacturer: Pharmaceutical Factory of Beijing Tongrentang Technology Development Co., Ltd.; NMPA approval no. Z11020054) for treatment, with 20 pills each time for water-honey pills and 1 pill each time for big candied pills and 2 times a day.

### 2.4. Observation Indexes

With the pathological examination results as the “gold standard,” the specificity, sensitivity, positive and negative detection results, and detection accuracy were compared between the two groups.

Specificity = the cases of negative diagnostic results detected by color Doppler ultrasound/the cases of actual negative results detected by color Doppler ultrasound × 100%; sensitivity = the cases of positive diagnostic results detected by color Doppler ultrasound/the cases of actual positive results detected by color Doppler ultrasound × 100%; detection accuracy = the cases of both positive and negative results detected by color Doppler ultrasound and pathological diagnosis/the total number of patients × 100% [13–15].

All patients with positive results were detected twice. If the results were the same as those of the first detection, they were judged to be positive. If the results were different from those of the first detection, the patients were detected for the third time, and the third detection results were the final results.

### 2.5. Statistical Treatment

The selected data processing software for this study was SPSS 20.0, and GraphPad Prism 7 (GraphPad Software, San Diego, USA) was used to draw the pictures of the data. Measurement data were expressed by ($\bar{x}$ ± *s*) and tested by *t*-test. Enumeration data were expressed as (*n* (%)) and tested by *X*^2^ test. The differences had statistical significance when *P* < 0.05.

## 3. Results

### 3.1. Comparison of Positive and Negative Detection Results between the Two Groups

The cases of both positive and negative detection results in the experimental group (39 and 9 cases) were significantly more than those in the control group (21 and 2 cases), with statistical significance (*P* < 0.05), as shown in Table 1.

### 3.2. Comparison of Specificity and Sensitivity between the Two Groups

**Figure 1:**
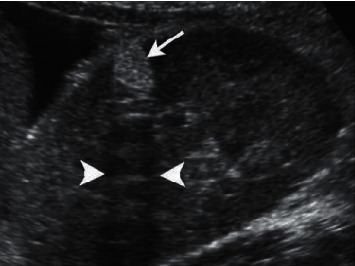
A detection image of positive tumors. *Note.* The black and white ultrasound image of the longitudinal section of the right kidney showed a cluster of echoes (as shown by the arrow) with a posterior sound shadow (as shown by the short arrow).

**Figure 2:**
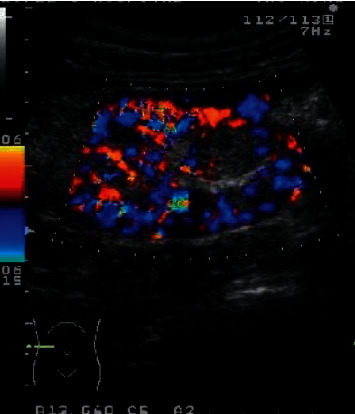
A detection image of negative tumors. *Note.* Yellow and blue represented different blood flow directions, in which the yellow part indicated the blood flow direction, while the blue part indicated the direction different from that of the blood flow.

**Figure 3:**
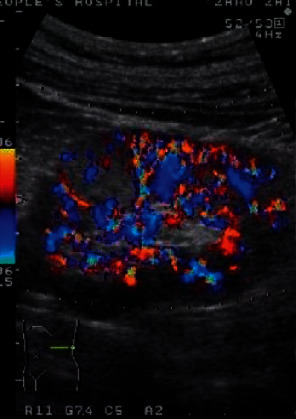
A detection image of negative tumors. *Note.* Yellow and blue represented different blood flow directions, in which the yellow part indicated the blood flow direction, while the blue part indicated the direction different from that of the blood flow.

Figure 1 shows the detection image of positive tumors in this study, and Figures 2 and 3 show the imaging images of negative tumors. The diagnostic specificity and sensitivity of the experimental group (90.00% and 97.50%) were significantly higher than those of the control group (18.18% and 53.85%), with statistical significance (*P* < 0.05), as shown in Table 2.

### 3.3. Comparison of Detection Accuracy between the Two Groups

**Figure 4:**
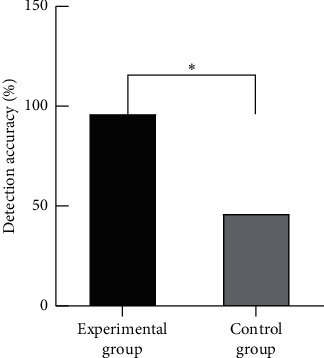
Comparison of detection accuracy between the two groups. *Note.* The abscissa represented the experimental group and control group, while the ordinate represented detection accuracy (%). ^*∗*^ denoted *P* < 0.05. ^*∗*^ represented the comparison of detection accuracy between the two groups. The detection accuracy was 96% in the experimental group and 46% in the control group, with statistical significance (*X*^2^ = 60.71 and *P* < 0.001).

The detection accuracy in the experimental group (96%) was significantly higher than that in the control group (46%), with statistical significance (*P* < 0.05), as shown in Figure 4.

## 4. Discussion

Small renal tumors including benign and malignant tumors refer to intrarenal tumors less than 3 cm in diameter, and common benign tumors include angiomyolipoma [16–19]. Besides, malignant renal tumors, also known as renal cell carcinoma, commonly include Wilms' tumors and transitional cell carcinoma. Compared with benign renal tumors, renal cell carcinoma has lower incidence but poses a greater threat to patients' physical health. The infiltrative range, treatment, and prognosis of the tumors are closely related to the early diagnosis, and thus, exploring effective diagnostic methods has always been the focus in the research field of small renal tumors. All patients in this study were diagnosed with small renal tumors by pathological examination. The results of different detection methods showed that color Doppler ultrasound combined with SMI was more accurate, with higher specificity, sensitivity, and detection accuracy. The principle of SMI is to suppress blood vessels by clutter, so as to extract low-speed blood flow signals from blood vessels and express them as color overlaying images or monochromatic blood flow images. This shows that SMI can detect more blood flow signals and is more sensitive compared with color Doppler ultrasound. The results of this study showed that color Doppler ultrasound combined with SMI can obviously improve positive and negative detection rates. The specificity, sensitivity, and detection accuracy in the experimental group were significantly higher than those in the control group, with statistical significance (*P* < 0.05), indicating that color Doppler ultrasound combined with SMI can improve the diagnostic accuracy in patients with small renal tumors, which is worthy of application and popularization. Scholar Zhou et al. [20] pointed out in their study that color Doppler ultrasound combined with SMI could improve the positioning accuracy of the perforator flap artery in patients with diabetes, which is consistent with the conclusion and proves the scientificity of results in this study.

At present, many scholars have affirmed the application value of color Doppler ultrasound in the diagnosis of small renal tumors in their research reports, but some clinical trials have revealed that the detection rate and accuracy of single color Doppler ultrasound for small renal tumors are not high, and its application effect remains unsatisfactory [21, 22]. SMI is an imaging test that mainly targets at blood flow velocity in microvessels as well as blood flow classification. In this study, the combination of SMI and color Doppler ultrasound was applied to the diagnosis of renal small tumors, further exploring the diagnostic effect, diagnostic specificity, sensitivity, and detection accuracy. Although this study confirmed the diagnostic value of color Doppler ultrasound combined with SMI in the detection of small renal tumors less than 3 cm treated with Jinkui Shenqi pills, its diagnostic efficacy was not absolute. In clinical practice, it is necessary to combine the results of urine routine, blood biochemistry, and blood tumor markers to further improve the diagnostic accuracy of the disease.

## 5. Conclusions

In conclusion, the detection mode of small renal tumors by color Doppler ultrasound combined with SMI can significantly improve the specificity, sensitivity, and detection accuracy. Therefore, color Doppler ultrasound combined with SMI has high application value and significant diagnostic effect, which is worthy of application and popularization in clinical practice.

## Figures and Tables

**Table 1:** Comparison of positive and negative detection results between the two groups.

Group	Cases of positive detection results	Cases of negative detection results
Experimental group	39^*∗*^	9^*∗∗*^
Control group	21	2
*X* ^2^	13.50	5.01
*P*	<0.001	0.03

*Note.*^*∗*^ indicated that the number of positive detection results was higher compared with the control group (*P* < 0.05). ^*∗∗∗*^ indicated that the number of negative detection results was higher compared with the control group (*P* < 0.05).

**Table 2:** Comparison of specificity and sensitivity between the two groups.

Group	Specificity	Sensitivity
Experimental group	90.00%^*∗*^	97.50%^*∗∗*^
Control group	18.18%	53.85%
*X* ^2^	103.86	51.75
*P*	<0.001	<0.001

*Note.*^*∗*^ indicated a significant difference in the specificity between the two groups (*P* < 0.05). ^*∗∗*^ indicated a significant difference in the sensitivity between the two groups (*P* < 0.05).

## Data Availability

The datasets used and/or analyzed during the current study are available from the corresponding author upon reasonable request.

## References

[1] Quader N., Hodgson A. J., Mulpuri K., Cooper A., Garbi R. (2021). 3-D ultrasound imaging reliability of measuring dysplasia metrics in infants. *Ultrasound in Medicine and Biology*.

[2] Delory T., Goujon A., Masson-Lecomte A. (2021). Fosfomycin-trometamol (FT) or fluoroquinolone (FQ) as single-dose prophylaxis for transrectal ultrasound-guided prostate biopsy (TRUS-PB): a prospective cohort study. *International Journal of Infectious Diseases*.

[3] Won J. Y., Kim D. K., Park S. Y. (2021). Renal mass cryoablation: melting time analysis of radiographic ice-ball after 5-minute active thawing by using serial ultrasound. *European Journal of Radiology*.

[4] Craver D., Ahmad A., Colclough A. (2020). 145Point of care lung ultrasound in patient triage: integration of ultrasound into a streaming pathway for COVID-19. *Emergency Medicine Journal*.

[5] Malik H., Appelboam A., Taylor G., Wood D., Knapp K. (2020). 71Ultrasound directed reduction of Colles’ type distal radial fractures in ED (UDiReCT): a feasibility randomized controlled trial. *Emergency Medicine Journal*.

[6] Haach V. G., Carrazedo R., Ribeiro P. O., Ferreira L. P. A., Abe P. (2021). Evaluation of elastic anisotropic relations for plain concrete using ultrasound and impact acoustic tests. *Journal of Materials in Civil Engineering*.

[7] Trujanovic R., Otero P. E., Larenza M. M. P., Gasparik K. N. (2020). Development of a lateral ultrasound-guided approach for the radial, ulnar, median and musculocutaneous (RUMM) nerve block in a calf undergoing surgical fixation of the antebrachium. *Veterinary Record Case Reports*.

[8] Cao D., Xu X., Jiang S. (2021). Ultrasound-electrochemistry enhanced flotation and desulphurization for fine coal. *Separation and Purification Technology*.

[9] Ye F.-Y., Lyu G.-R., Li S.-Q. (2021). Diagnostic performance of ultrasound computer-aided diagnosis software compared with that of radiologists with different levels of expertise for thyroid malignancy: a multicenter prospective study. *Ultrasound in Medicine and Biology*.

[10] Jamil L. A., Sami H. Z., Aghaei A., Moinfar S., Ataei S. (2021). Combination of modified ultrasound-assisted extraction with continuous sample drop flow microextraction for determination of pesticides in vegetables and fruits. *Microchemical Journal*.

[11] Bengtson B. P. (2021). Instructional course: officed-based high-resolution ultrasound for the plastic surgeon. *Clinics in Plastic Surgery*.

[12] Nazer B., Giraud D., Zhao Y. (2021). Microbubble-facilitated ultrasound catheter ablation causes microvascular damage and fibrosis. *Ultrasound in Medicine and Biology*.

[13] Surya Y., Christopher G., Raquel T. (2021). A rare diagnosis of segmental testicular infarction on colored Doppler ultrasound. *Visual Journal of Emergency Medicine*.

[14] Abou-Zeid S. M., Ahmed A. I., Awad A. (2021). Moringa oleifera ethanolic extract attenuates tilmicosin-induced renal damage in male rats via suppression of oxidative stress, inflammatory injury, and intermediate filament proteins mRNA expression. *Biomedicine & Pharmacotherapy*.

[15] Kim J. H., Shim S. R., Lee H. Y. (2020). Prevalence of benign pathology after partial nephrectomy for suspected renal tumor: a systematic review and meta-analysis. *International Journal of Surgery*.

[16] Li C. C., Chien T. M., Huang S. P. (2020). Single-site sutureless partial nephrectomy for small exophytic renal tumors. *Journal of Clinical Medicine*.

[17] Ficarra V., Caloggero S., Rossanese M. (2020). Computed tomography features predicting aggressiveness of malignant parenchymal renal tumors suitable for partial nephrectomy: a review. *Minerva Urologica e Nefrologica*.

[18] Masood Y., Hussain I., Khan U. U., Khalid M. U., Javed M. U. (2020). Acute Lobar Nephronia in an infant presented as a renal tumor. *Urology Case Reports*.

[19] Gavens E., Arul G. S., Pachl M. (2020). A single centre matched pair series comparing minimally invasive and open surgery for the resection of pediatric renal tumours. *Surgical Oncology*.

[20] Zhou Y., Liu J., Yue Q. (2020). Color Doppler ultrasound combined with contrast-enhanced ultrasonography and ultrasound microangiography in the arterial location of perforator flap in diabetic patients. *Chinese medical device information*.

[21] Kobari Y., Takagi T., Yoshida K., Ishida H., Tanabe K. (2021). Comparison of postoperative recovery after robot‐assisted partial nephrectomy of T1 renal tumors through retroperitoneal or transperitoneal approach: a Japanese single institutional analysis. *International Journal of Urology*.

[22] Li Y., Liu X., Duan C.-f., Zhuang X.-h., Ge W., Song X.-f. (2021). Imaging manifestations of congenital mesoblastic nephroma. *Clinical Imaging*.

